# Nitrate of Potass in Hæmoptysis

**Published:** 1842-08-27

**Authors:** 


*Nitrate of Potass in Hæmoptysis.*—M. Gaudinau has employed large doses of nitrate of potass in many cases of hæmoptysis, with great success. He prescribes from eight to fifteen scruples of nitrate of potass, dissolved in one hundred and twenty of gum water, and sweetened with fifteen of syrup, one or two of these mixtures being administered in the course of the twenty-four hours. He has never found any unpleasant consequences in the digestive apparatus from so large a dose. This remedy has succeeded in cases of frightful hæmorrhage, when repeated general and local bleedings, pectoral ptisans, opiate looches, revulsives, and astringents have failed. The nitrate of potass has failed in only two cases out of fifty.—*Ibid, from Travaux de la Société Méd. de Dijon.*

